# Femoral Artery Injuries in Closed Femur Shaft Fractures: Case Report

**DOI:** 10.1055/s-0042-1756206

**Published:** 2022-10-13

**Authors:** Sathya Vamsi Krishna, Sindhu B., Suhas T.R, Chandrashekar H. Sumanahalli

**Affiliations:** 1 Department of Microvascular and Orthopaedic Surgery, Sanjay Gandhi Institute of Trauma and Orthopedics, Bengaluru, Karnataka, India

**Keywords:** closed femur shaft fracture, vascular injury, superficial femoral artery, thrombectomy, fasciotomy, intramedullary interlocking nail

## Abstract

**Case 1 and 2** Two young male patients, sustained injury to the superficial femoral artery (SFA) following a closed femur shaft fracture. The arterial injuries were confirmed by computed tomography angiography and both underwent fracture fixation and on SFA exploration; a thrombosed arterial segment was noted at the fracture site, addressed with arteriotomy and thrombectomy to restore the vascularity. At 1-year follow-up, both patients had good union at the fracture site and a well-perfused limb.

**Conclusion** Thorough clinical examination and appropriate diagnostic studies can diagnose these rare vascular injuries in closed fractures and with early vascular repair potentially limb-threatening complications can be prevented.

Any blunt injury, if severe enough to cause a bony injury, has the potential to cause concomitant arterial injury. Arterial injury can vary, from traumatic laceration to thrombosis and, if not intervened upon early can lead to prolonged ischemia, leading to potentially devastating complications including the need for an amputation. 1 Most of the encountered arterial injuries with fractures are due to penetrating, crush injuries leading to a definitive compound/open fractures. As such, closed injuries presenting with an arterial injury are rare and hence the primary surgeon may not suspect it. In this report, we present two cases of superficial artery injuries associated with closed femoral shaft fractures.

## Case Report

### Case 1

**Fig. 1:**
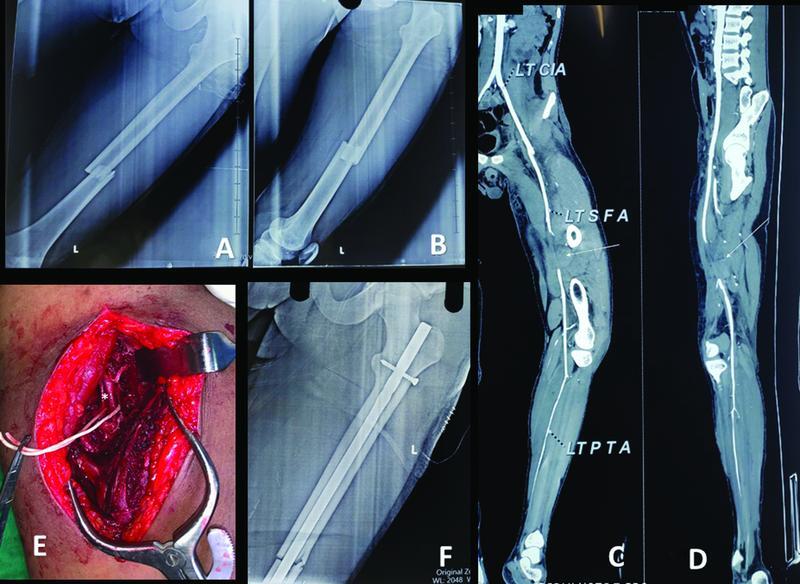
( **A** , **B** ) Anteroposterior/lateral view radiographs of the left femur showing transverse midshaft femur fracture. ( **C** , **D** ) Computerized tomography angiography image showing interruption of the superficial femoral artery at the level of fracture site. ( **E** ) Intraoperative image of superficial femoral artery showing thrombus at the level of fracture site. ( **F** ) Postoperative radiograph showing reduced femur shaft fracture with intramedullary interlocking nail in situ.

A 22-year-old male patient sustained an injury to his left thigh when he met with a road traffic accident after being hit by a truck. The patient presented 6hours after the injury. On examination, the patient was conscious and oriented, hemodynamically stable. Clinical examination revealed swollen and tense left thigh with absent distal pulses. Limb oxygen saturation was not recordable. The patient had toe and ankle movements with no other signs of ischemia. Radiographs showed transverse left midshaft femur fracture ( Fig. 1A, B ). Computed tomography angiography (CTA) revealed complete interruption of superficial femoral artery (SFA) at the level of fracture secondary to an occluding thrombus ( Fig. 1C, D ).

The patient was taken for surgery on an emergent basis. First, the fracture was stabilized with an intramedullary interlocking nail. No circulatory improvement noted after the procedure. Vascular exploration was done on the medial side of the thigh at the level of fracture between the adductor and extensor compartment of the thigh, which revealed a thrombosed femoral artery segment of 5cm with surrounding hematoma and no transmitted pulsations ( Fig. 1E ). A small arteriotomy of 5mm was done just proximal to the level of the thrombus artery using a scalpel no. 11 and thrombectomy was done proximally and distally with a Fogarty's catheter. Immediately after the thrombectomy, a sudden gush of fresh blood was noted from the arteriotomy site. Vascular clamps were applied proximal and distal to the arteriotomy site. Each clamp was released individually to check the anterograde and reverse flow. The reason for the arterial thrombosis is attributed to a minor tear in the artery due to the fracture fragments which lead to hematoma around the artery and thrombosis of the vessel. The arteriotomy site and the tear on the vessel were well irrigated with heparin saline and adventitia was debrided, and the opening was repaired with Prolene 7–0. After the repair, the Acland clamp test was performed to check the anterograde and reverse flow. 2

Within a few minutes both distal pulses were well palpable and oxygen saturation was restored. Intraoperatively patient was given heparin 5,000IU bolus dose and postoperatively patient was put on low molecular weight heparin 60mg subcutaneously twice a day for 5 days and discharged with an advice of tablet aspirin 75mg once a day for 3 months. Follow-up was uneventful and weight-bearing was started at 6 weeks and bone showed good union with no long-term signs of ischemia. A repeat Doppler study done at 1 year showed good triphasic flow of the femoral artery.

### Case 2

**Fig. 2:**
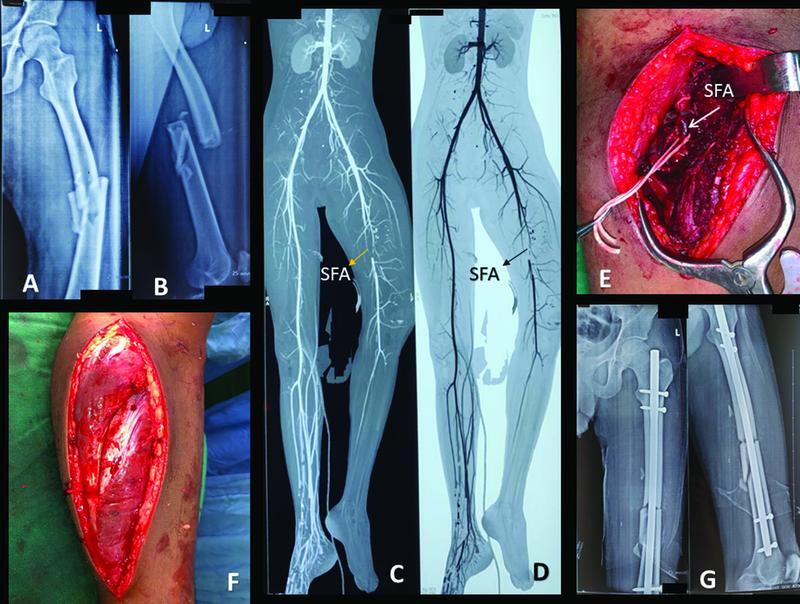
( **A** , **B** ) Anterioposterior and lateral radiograph showing comminuted midshaft femur fracture. ( **C** , **D** ) Computerized tomography angiography image showing interruption of superficial femoral artery at the level of fracture site. ( **E** ) Intraoperative image of superficial femoral artery showing thrombus at the level of fracture site. ( **F** ) intraoperative image showing fasciotomy over lateral aspect of leg. ( **G** ) Postoperative radiograph showing reduced femur shaft fracture with intramedullary interlocking nail in situ.

A 21-year-old male patient was referred to our department with an alleged history of a motor vehicle accident 4 days back due to falling from a bike. After the injury, he was taken to the primary care center where he was diagnosed with diffuse axonal brain injury along with left pulseless limb and associated femur shaft fracture. In view of his brain condition, the fractured limb was immobilized with a Thomas splint and not intervened early. However, he had already undergone a CTA of the left limb and diagnosed with SFA injury. On presentation to our center, patient's Glasgow Coma Scale was 5/15 but was hemodynamically stable. On local examination, there was a deformed left thigh with absent peripheral pulses and unrecordable oxygen saturation on the affected limb. Active foot movements could not be assessed because of poor GCS; however, his limb was warm, in good color, and soft calf muscles with unlikely signs of ischemia. Radiograph of left thigh revealed left comminuted midshaft femur fracture ( Fig. 2A, B ). CTA performed earlier showed complete interruption of SFA at the level of fracture ( Fig. 2C, D ).

Similar to the previous case the fracture was stabilized with an intramedullary interlocking nail. As there was no circulatory improvement after closed reduction, femoral vessels were explored, and the intraoperative finding was similar to the previous case ( Fig. 2E ). Unlike the first case, the arteriotomy was done distal to the thrombus and clots were removed with Fogarty's catheter. Anterograde and reverse flow were confirmed and arteriotomy site along with the tear on the vessel was closed with Prolene 7–0. The peripheral pulses were restored immediately. Prophylactic fasciotomy of the left leg was done through both medial and lateral incision decompressing all four compartments keeping in view the delayed presentation and unreliability of assessing compartment syndrome postoperatively considering poor GCS ( Fig. 2F ). Anticoagulation protocol similar to the previous case was followed. Successively patient was intubated for airway protection. He underwent one stage of debridement for the fasciotomy site on day 2 followed by split skin thickness graft cover on day 5. The patient stayed in the intensive care unit for more than 20 days due to the head injury. After the recovery patient underwent rehabilitation and discharged with oral anticoagulants for 3 months similar to the previous case. On subsequent follow-up, he had persistent foot drop and decreased sensation of the foot over the supply of deep peroneal and posterior tibial nerve areas but mobilized with a splint. At 1 year, his fracture united well and arterial Doppler showed a triphasic flow of the femoral artery; however, his limb weakness persisted because of ischemic insult to the muscles due to delayed presentation. He is waited for further corrective surgeries of the foot.

## Discussion

Arterial ischemia in lower extremity injuries vary from intimal tear/contusion with subsequent secondary thrombosis, to occlusion due to extrinsic compression, angulation, dissection with an intact adventitia, spasm, or entrapment of the vessels within fracture ends. 3 Of all the vessels, femoral arterial injuries constitute 70% of all vascular injuries, and 90% of these is due to penetrating trauma. 4 Arterial injuries lead to thrombus within the vessel or rent in the vessel can cause a false aneurysm. A false aneurysm is formed through a full-thickness defect in the vessel leading to the escape of blood into periarterial tissue. This tissue along with the thrombus form the wall of the aneurysm and the blood enters this cavity during systole leading to an expanding pulsatile lesion with a systolic bruit. 5 There are few case reports describing femoral artery aneurysms associated with femoral shaft fractures. 5 6 Blunt arterial injury is rare and reported in 0.3% of long bone fractures. 3 7 8 Blunt vascular injury prognosis is worse than penetrating injury and has a higher risk of amputation because the blunt injury is associated with injury to surrounding structures which include bone, soft tissue, and nerve complicating the management. 3 7 9 10 11 Arterial repair done in vascular injuries reduced the risk of amputation from 49.6 to 10.8%. 12 13

In closed femoral fractures, arterial injures are overlooked, because it is uncommon and in false aneurysms, the distal pulses being normal can be misleading, and when in doubt further imaging is recommended. 5 8 Doppler is a good diagnostic tool and if doubt exists, arteriography (arterial angiogram) is recommended. The strong indications for arteriography or surgical exploration include excessive bleeding, expanding hematoma, bruit or thrill over injured vessels, and absent pulses. 9 14 Digital subtraction angiography remains the gold standard. Arteriography can establish the exact location, extent of arterial damage, or extrinsic cause for pulselessness, on the contrary, it is time-consuming. 3

Safe time for surgical intervention is considered between 6 and 8hours 13 ; however, adequate collateral circulation provides a longer safe period. 15 In our second case though the presentation was late, considering the collaterals, limb condition (soft and warm), and muscle texture during fasciotomy we proceeded with revascularization. However, delayed repairs can end up with poor functional outcome such as contracture or atrophy similar to our case. 15 Patients with prolonged ischemia have a risk of renal failure due to increased levels of myoglobin and its products which can be avoided with good hydration and mannitol preoperatively, intraoperatively, and postoperatively. 3 Due to delayed presentation in our second patient, to avoid renal complications, we infused injection mannitol 100mL thrice a day for 5 days from the day of arrival to our hospital and hydrated with fluids 100mL per hour for 7 days. Isolated vascular injuries with good collaterals could have been ignored but it was intervened in our case as it was performed in the same procedure along with femur fracture fixation just to avoid further compromise with collaterals after fixation. Relative indication is to avoid complications such as arteriovenous fistula/pseudoaneurysm and prevent ischemic muscle pain in future.

For fractures with arterial injury, primary arterial repair should always be accompanied by internal fixation for closed fractures or with clean wounds, and external fixator or traction for contaminated or delayed presented injuries. 16 17

Various treatment options include lateral repair, venous patch graft, resection of injured vessel and primary repair, autogenous or synthetic vein graft interposition, bypass graft, or thrombectomy by Fogarty's catheter. 3 Results with interposition veins graft have been good. 18 Synthetic arteries have a higher risk of false aneurysms. 19 Endovascular management in peripheral vascular injuries is limited. 20 In our series, we were able to get away with a simple thrombectomy using Fogarty's catheter to establish the circulation accompanied by internal fixation using an intramedullary interlocking nail. Vein graft was not considered in our cases since the injured vessel had a good vessel wall with minimal intimal damage.

Compartment syndrome is a known complication associated with vascular injuries due to reperfusion injury. In our second case, prophylactic four-compartment fasciotomy was done keeping in view the delayed presentation in addition to the reperfusion.

Severely damaged or ischemic limb resulting in nonfunctional or useless limb should undergo primary amputation. 3 Failure of prompt diagnosis of vascular injury and the severity of the soft tissue damage are the potential reasons for the amputation. Closed fractures can be accompanied by vascular injury where its early diagnosis with the help of a CTA and speedy surgical intervention to restore the blood supply can attain an excellent functional limb. It is recommended to follow strict immobilization of the injured limb for at least 6 weeks to prevent unnecessary insult to the artery at the fracture site.
